# Zinc(II) Complexes with Dangling Functional Organic Groups

**DOI:** 10.1002/ejic.201200558

**Published:** 2012-08-16

**Authors:** Jingxia Yang, Michael Puchberger, Renzhe Qian, Christian Maurer, Ulrich Schubert

**Affiliations:** [a] Institute of Materials Chemistry, Vienna University of Technology1060 Wien, Austria; [b] Institute of Organic Chemistry, University of Vienna1090 Vienna, Austria

**Keywords:** Coordination polymers, Zinc, Carboxylate ligands, Schiff bases

## Abstract

Zinc(II) complexes with dangling functional organic groups were synthesized by reaction of zinc acetate with a series of bifunctional *p*-substituted benzene derivatives (a combination of carboxylate, oximate, amino, β-ketoimine, and salicylaldime groups). Selective coordination to carboxylate groups was observed when the second functional group was an oxime or β-ketoimine group. When the second group was an amine or salicylaldimine moiety, these groups were additionally coordinated. From the reaction with *p*-aminobenzoic acid, the compound [Zn_2_(OOCCH_3_)(OOC–C_6_H_4_–NH_2_)_3_]_∞_ was crystallized. It is a three-dimensional coordination polymer with bridging aminobenzoate ligands.

## Introduction

**Scheme 1:**
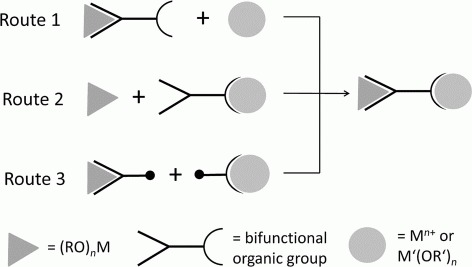
Synthesis routes for bimetallic precursors with organic linkers.

Single-source precursors are mainly used in chemical vapor deposition and sol–gel processing to introduce two different elements into the final material. Typical single-source precursors for sol–gel processing are bimetallic alkoxides in which the two metals are bridged by alkoxo groups, which are then lost upon sol–gel processing (i.e., upon formation of mixed-oxide systems).^1^ An unexploited possibility is the option to utilize single-source precursors to control the spatial arrangement of two components in the final material. This requires a different composition of the bimetallic precursor. Our approach is to link the individual metal components through organic groups. Possible synthesis routes of such precursors are shown in Scheme 1.

Silica-based mixed-metal oxides are mostly prepared by route 1 because of the availability of a variety of alkoxysilanes (RO)_3_Si(CH_2_)_3_X, in which X is a coordinating group (such as an amino^2^ or β-diketonate group^3^). When (RO)_3_Si(CH_2_)_3_X is treated with metal ions or metal alkoxide moieties (M), a bimetallic precursor [(RO)_3_Si(CH_2_)_3_X]*_n_*M is formed. Sol–gel processing of such precursors was used to prepare nanocomposite materials.^4^ A recent example of route 3 (the coupling of two ligands through appropriate organic groups) is the coupling of 3-isocyanatopropyltriethoxysilane with metal hydroxyacetates.^5^

A generalization of this method for the preparation of mixed oxides of any two metallic elements is less straightforward. Routes 1 and 2 require bifunctional organic compounds X′–Y–X with two different coordinating groups (X and X′, Y = inert spacer). The challenge is that the functional groups must selectively react with just one metal component [i.e., coordination of either metal to both coordination groups (which would lead to coordination polymers) must be avoided].

We have previously given an example for route 1 by coordinating lysine to titanium and zirconium alkoxides (chelate formation by the carbonyl oxygen atom and the α-amino group) followed by the coordination of metal ions to the dangling ω-amino group.^6^ The use of lysine cannot be generalized, however, because substitution of the metal alkoxides competes with lactam formation.^7^

In this paper, we investigate the selectivity issue for Zn^2+^ ions (for proof of principle) by using various bifunctional *p*-substituted benzene derivatives and report the synthesis of complexes Zn(X′–Y–X)_2_. In a follow-up paper, we will report on the following steps, specifically the coordination of Ti(OR)_4_ to the dangling group X according to route 2, and sol–gel processing of the single-source precursor.^8^ Benzene derivatives were chosen to avoid backbiting of the ligand, and the dangling groups X according to their ability to coordinate to metal alkoxide moieties.^9^ The complex Zn(PSBO)_2_ (PSBO-H = salicyliden-*p*-aminoacetophenone oxime), which has a dangling oxime functionality, was previously reported^10^ but is included here for comparison. Complexes M′–X′–Y–X are not only suitable building blocks for the preparation of mixed oxides by following route 2, but might also be useful as a new type of metal–organic linker for metal–organic framework (MOF) structures.

The structures of zinc complexes are well documented (see the literature^11^ for classification and analysis of structural data). Therefore, this article does not deal with new structures, but instead concentrates on testing the possibility of selective coordination of bifunctional ligands.

## Results and Discussion

**Scheme 2:**
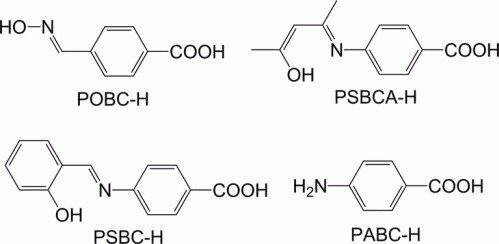
Benzoic acid derivatives used as ligands.

Given the high tendency of Zn^2+^ to form carboxylate complexes, four benzoic acid derivatives with a second functional group capable of coordinating to alkoxide moieties in the *para* position were initially chosen, namely, *p*-carboxybenzaldehyde oxime (POBC-H), *p*-[(3-hydroxy-1-methyl-2-butenylidene)amino]benzoic acid (PSBCA-H), *p*-salicylidene-*p*-aminobenzoic acid (PSBC-H), and *p*-aminobenzoic acid (PABC-H) (Scheme 2).

The ^1^H NMR spectrum of POBC-H showed two signals in the region of acidic protons (Figure S1 in the Supporting Information). The heteronuclear multiple-bond correlation (HMBC) spectrum allowed us to assign the peak at *δ* = 12.99 ppm to COO*H* and the peak at *δ* = 11.52 ppm to NO*H* (Figure S2 in the Supporting Information). After reaction of zinc acetate with two molar equivalents of POBC-H, the signal for COO*H* had disappeared, and the aromatic protons *ortho* to the COO group had shifted from *δ* = 7.71 to 7.63 ppm. Furthermore, the *C*OO signal in the ^13^C NMR spectrum was shifted from *δ* = 166.9 to 171.1 ppm (Figure S1 in the Supporting Information). The NMR spectroscopic data thus indicated coordination of the COO group to Zn^2+^. The signal of the NO*H* group in the ^1^H NMR spectrum was also slightly shifted, from *δ* = 11.52 to 11.37 ppm, but the *C*=N signal in the ^13^C NMR spectrum was not, which means the NOH was not coordinated. The absence of signals for acetate groups in the NMR spectra indicated that acetic acid, formed by the substitution reaction, was removed during the workup procedure.

FTIR measurements confirmed these results. In the spectrum of POBC-H (Figure 1, a), the bands at 1685, 1611, 1290, and 988 cm^–1^ correspond to ν_C=O_, ν_C=N_, ν_C–O_, and ν_N–O_, respectively. After coordination to Zn^2+^ (Figure 1, b), the ν_C=O_ band was shifted from 1685 to 1549 cm^–1^ and that of ν_C–O_ from 1290 to 1410 cm^–1^. In contrast, the bands of ν_C=N_ and ν_N–O_ shifted only slightly, from 1611 to 1602 cm^–1^ for ν_C=N_ and from 988 to 979 cm^–1^ for ν_N–O_.

**1:**
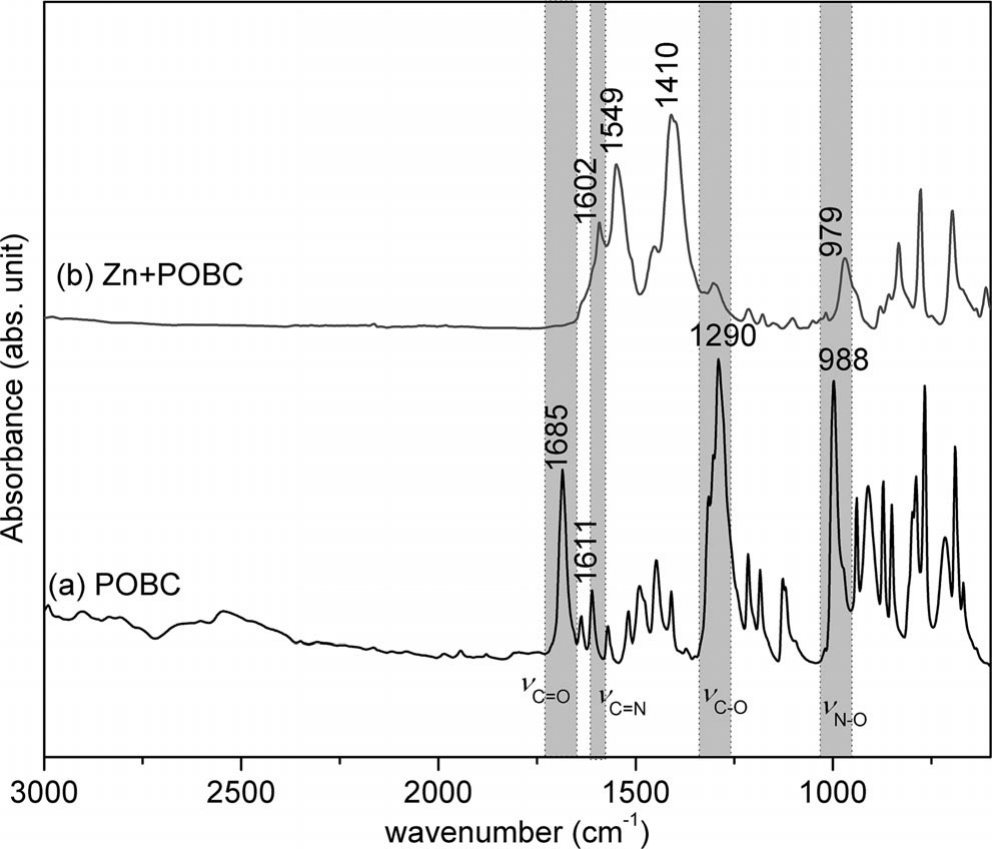
FTIR spectra of POBC-H and Zn(POBC)_2_.

Similar results were obtained for PSBCA-H. The appearance of two OH signals in the ^1^H NMR spectrum confirmed that the enol–imine form is the predominant tautomer in the liquid phase (Figure S3 in the Supporting Information), as previously reported.^12^ In the crystalline state, the keto–enamine tautomer was found.^12^ An HMBC spectrum (Figure S4 in the Supporting Information) showed that the resonance at *δ* = 12.81 ppm can be assigned to COO*H* and the resonance at *δ* = 12.61 ppm to the enol O*H* group. After reaction with half a molar equivalent of zinc acetate, the resonance of the COO*H* proton had disappeared, whereas the other resonance was not significantly shifted. In the ^13^C NMR spectra, the *C*OO resonance was shifted from *δ* = 166.72 to 170.87 ppm, whereas that of C=*C*(Me)OH was slightly shifted from *δ* = 142.69 to 140.95 ppm. The other signals were almost unchanged.

PSBCA-H and the derived Zn complex were also characterized by FTIR (Figure S5 in the Supporting Information). The absorption bands of PSBCA-H for ν_COO_, ν_C=N_, ν_C–O_, and ν_C–N_ are at 1695, 1595, 1286, and 1025 cm^–1^, respectively. After reaction with Zn^2+^, ν_COO_ shifted to 1579 cm^–1^ and ν_C–O_ to 1314 cm^–1^. The bands for ν_C=N_ and ν_C–N_ were unchanged at 1598 and 1026 cm^–1^. The NMR and FTIR spectra indicated that Zn^2+^ was coordinated to the carboxylate group and that the Schiff base moiety was unchanged.

**Scheme 3:**
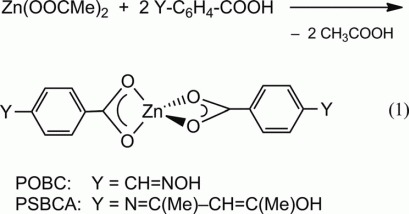
Suggested structure of Zn(POBC)_2_ and Zn(PSBCA)_2_.

Based on the NMR and IR spectroscopic results, the structures shown in Scheme 3 are proposed for Zn(POBC)_2_ and Zn(PSBCA)_2_, in which the COO groups are coordinated to Zn and the Y groups are dangling. This composition was also supported by elemental analyses and TGA measurements (by which the ZnO proportion is determined after burning off all organic components) (Figures S6 and S7 in the Supporting Information).

Somewhat different results were obtained for PSBC-H, a carboxylate-substituted salicylaldimine. According to ^1^H and ^13^C NMR spectroscopic data (Figures S8 and S9 in the Supporting Information), the coordination situation of PSBC is more complex. When the employed Zn(OAc)_2_/PSBC ratio was 1:2 as before, the NMR spectra showed that the COO*H* signal at *δ* = 12.97 ppm had disappeared after the reaction, thus indicating that the carboxylate group was coordinated to Zn^2+^. This was confirmed by ^13^C NMR spectroscopy, in which the *C*OO signal shifted from *δ* = 166.82 to 170.98 ppm. Although there was only one signal for the *C*OO group in the ^13^C NMR spectrum of the obtained Zn–PSBC complex, there were two signals for salicylaldimine moieties. The first half of the groups were not coordinated. The ^1^H NMR spectroscopic signal for the OH group shifted from *δ* = 12.71 to 12.89 ppm, and integration of the both the O*H* and N=C*H* signal (at *δ* = 9.0 ppm) was reduced to 50 %. The ^13^C NMR spectroscopic signal of the *C*–OH carbon was not shifted. The second half of the salicylaldimine moieties was coordinated to Zn^2+^. A second N=C*H* signal was observed at *δ* = 8.71 ppm with 50 % intensity. Furthermore, part of the *C*H=N signal was shifted from *δ* = 164.20 to 168.88 ppm.

When the experiment was repeated with a Zn(OAc)_2_/PSBC-H ratio of 1:3, the chemical shifts in the ^1^H NMR spectrum were almost the same as for the 1:2 reaction. When both compounds were treated in a 1:1 ratio, the signal for OH was also present in the ^1^H NMR spectrum, but with a decreased intensity. This indicated that both the COOH and salicylaldimine group of PSBC can coordinate, but that coordination of COO was preferred.

IR spectra confirmed the NMR spectroscopic results. The spectrum of PSBC-H shows the stretching vibrations of C=O, C=N, COO, and C_Ph_–O at 1677, 1621, 1317, and 1283 cm^–1^, respectively. After reaction of PSBC-H with Zn(OAc)_2_, the IR spectra were almost the same for the three samples prepared with different Zn/PSBC-H ratios (Figure S10 in the Supporting Information). The C=O band shifted from 1677 to 1590 cm^–1^ and that of COO from 1317 to 1405 cm^–1^. The bands for C=N and C_Ph_–O appeared at the same wavenumber as that of PSBC-H, but with decreased intensity. Elemental analysis of the resulting solid showed that the carbon content was slightly higher than calculated for Zn(PSBC)_2_. Together with the spectroscopic results this might indicate that the solid could be a mixture of compounds with different coordination of the zinc ions. The important information in the context of this work, however, is that there is no coordination selectivity between the carboxylate and salicylaldimine group.

Zn(PABC)_2_ was previously prepared from a 1:2 ratio of Zn(NO_3_)_2_**·**6H_2_O and PABC-H.^13^ Its structure is a three-dimensional coordination polymer with PABC as a bridging ligand. Each zinc atom is coordinated by two NH_2_ groups and three oxygen atoms (one of a bridging and two of a chelating carboxylate group). Formation of a 3D network structure was also observed when Zn(NO_3_)_2_**·**6H_2_O was treated with 4-tetrazolyl benzene carboxylate.^14^

The spectra obtained by treating Zn(OAc)_2_ with PABC-H in a 1:2 molar ratio were not very meaningful. When a 1:4 ratio was employed, however, colorless crystals were obtained after 4 weeks. The COO*H* signal in the ^1^H NMR spectrum disappeared and that of NH_2_ shifted from *δ* = 5.87 to 5.51 ppm. This indicated that both NH_2_ and COO were coordinated to Zn^2+^. A single-crystal X-ray structure analysis showed that a three-dimensional coordination polymer with the composition [Zn_2_(OAc)(OOC–C_6_H_4_–NH_2_)_3_]_∞_ was formed (Figure 2).

**2:**
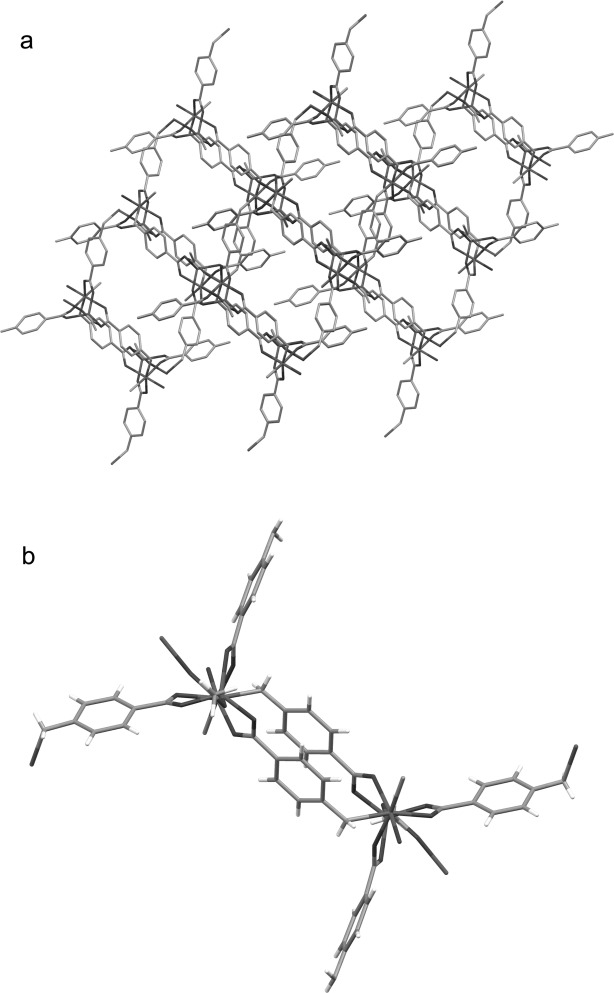
(a) Crystal structure of [Zn_2_(OAc)(OOC–C_6_H_4_–NH_2_)_3_]_∞_. (b) Section from the coordination polymer with four zinc atoms (two Zn atoms are pairwise located behind one another) showing the arrangement of the PABC ligands. The acetate ligands are perpendicular to the plane of the drawing (linear arrangement with the Zn–Zn axis).

In the crystal structure of Zn_2_(OAc)(PABC)_3_, each PABC molecule is coordinated to one zinc atom through the amino group and to two other zinc atoms through the bridging carboxylate group. The acetate ligands are bridging–chelating (i.e., the carboxylate group chelates one zinc atom, whereas one of the cyrboxylate oxygen atoms bridges two zinc atoms). There are two kinds of differently hexacoordinated zinc atoms. One is coordinated by the oxygen atoms of three bridging PABC ligands [in a *mer* arrangement, O–Zn–O 96.0(1)–100.6(1)°], two amino groups, and one oxygen of an acetate ligand. The other type of zinc atoms is also coordinated by the oxygen atoms of three bridging PABC ligands [in a *mer* arrangement, O–Zn–O 111.4(2)–118.8(1)°] in addition to one amino group and the chelating acetate ligand.

The results with the functional carboxylic acids POBC-H, PSBCA-H, PSBC-H, and PABC-H showed a clear preference for the coordination of the carboxylate group to Zn^2+^, a weaker tendency to coordinate salicylaldimine or amino groups, and no coordination of oximate or β-ketoiminate groups. This order of coordination ability was cross-checked for a series of substituted methylphenyl ketoximes. Oximate groups are especially suitable for the coordination of metal alkoxide moieties.^15^

**Scheme 4:**
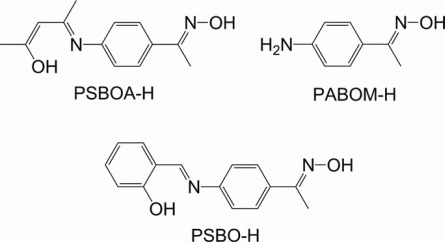
Other benzene derivatives used for reaction with Zn(OAc)_2_.

No differences were observed in the NMR spectra of PABOM-H or PSBOA-H (Scheme 4) solutions before and after the addition of zinc acetate. This indicates that no coordination occurred. Apparently, Ph–NH_2_ groups can only coordinate to Zn^2+^ in a supportive manner, as observed for Zn_2_(OAc)(PABC)_3_ or Zn_2_(PABC)_2_.

**Scheme 5:**
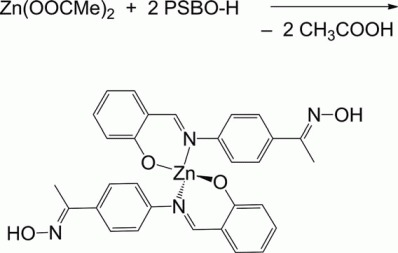
Suggested structure of Zn(PSBO)_2_.

The complex Zn(PSBO)_2_ was already described by Canpolat et al. (Scheme 5).^10^ The spectroscopic data are included here to illustrate the coordination selectivity of Zn^2+^ ions and because the assignment of the NMR spectroscopic signals is slightly different to what has been reported.

When PSBO-H was treated with Zn(OAc)_2_, the resonance for C–O*H* at *δ* = 13.03 ppm disappeared and that of N=C*H* shifted from *δ* = 8.98 to 8.80 ppm, whereas the signal of N–O*H* at *δ* = 11.26 ppm was nearly unchanged (Figures S11 and S12 in the Supporting Information). In the ^13^C NMR spectra, the imine *C*=N signal shifted from *δ* = 163.4 to 170.8 ppm and that of *C*–O from *δ* = 160.3 to 170.1 ppm. In the FTIR spectra (see Figure S13 in the Supporting Information) the stretching vibrations of C=N_imine_, C=N_oxime_, C–O, and N–O were observed at 1615, 1592, 1271, and 1002 cm^–1^ for PSBO-H. In the Zn–PSBO complex, these bands were at 1604, 1589, 1293, and 1006 cm^–1^. All spectroscopic data, as well as elemental analysis and TGA data (Figure S14 in the Supporting Information), support the exclusive coordination of the salicylaldimine group and formation of the complex Zn(PSBO)_2_ (Scheme 5).

## Conclusion

Reaction of various bifunctional *p*-substituted benzene derivatives with zinc acetate showed that selective coordination of one of the functional groups is possible, whereas the second, noncoordinated functional group is still available for the coordination of another metal moiety. Among the investigated functional groups, the preference for coordination to Zn^2+^ is –COOH < salicylaldimine < –NH_2_ << oxime, β-ketoimine. We found no indication for an interaction of the latter two functional groups with Zn^2+^. During the reaction, the acetate ligands of the employed zinc salt must be replaced by the corresponding group. There is no correlation between the coordination preference and the acidity of the corresponding groups. Thus, steric effects and the metal–ligand bond strength appear to be additional parameters that influence the substitution of the acetate groups.

The coordination selectivity allowed us to prepare the complexes Zn(X′–Y–X)_2_ (X = dangling organic group) with X′ = COO and X = oxime or β-ketoimine, as well as X = salicylaldiminate and X = oxime. The combination of carboxylate groups with salicylaldimine or amino groups resulted in complexes in which both groups were coordinated.

Dangling oxime groups are especially interesting because they have been proven to react with metal alkoxides and form stable oximate-substituted metal alkoxide derivatives.^15^ Reaction of the Zn(POBC)_2_ complex with Ti(OR)_4_ to give a bimetallic Zn/Ti single-source precursor and its use to prepare ZnO/TiO_2_ mixed oxides will be reported in a follow-up article.^8^

## Experimental Section

**Materials:** Zinc acetate dihydrate, *p*-carboxybenzaldehyde, sodium acetate, hydroxylamine hydrochloride, *p*-aminoacetophenone, *p*-aminobenzoic acid (PABC-H), salicylaldehyde (2-hydroxybenzaldehyde), and acetylacetone were used as received. The solvents used in the reactions were of analytical reagent grade and dried using standard procedures.

**Measurements:** ^1^H and ^13^C solution NMR spectra were recorded with a Bruker AVANCE 300 DPX (300.13 MHz for ^1^H, 75.47 MHz for ^13^C) equipped with a 5 mm broadband-inverse probe head and a *z* gradient unit. Infrared spectra were recorded with a Bruker Tensor 27 working in ATR Micro Focusing MVP-QL with a ZnSe crystal, and by using OPUS software version 4.0 for analysis. Thermogravimetric analyses (TGA) were performed with a Netzsch Iris TG 209 C instrument in a platinum crucible under synthetic air with a heating rate of 10 °C min^–1^ from 40 to 900 °C. The theoretical values for the elemental analyses were calculated for the water-free complexes; minor deviations of the found values therefore may be due to small proportions (less than one equivalent) of water.

***p*-Carboxybenzaldehyde Oxime (POBC-H):** *p*-Carboxybenzaldehyde (4.5 g, 30 mmol) and NH_2_OH**·**HCl (3.13 g, 45 mmol) was dissolved in ethanol (90 mL) and then mixed with NaOAc (3.7 g, 45 mmol) in water (270 mL). The solution was heated at reflux for 5 h. The solution was then extracted three times by ethyl acetate (EtOH/ethyl acetate ratio 1:4), once with brine, and dried with Na_2_SO_4_. Evaporating the solvent from the mixture of EtOH and ethyl acetate under reduced pressure resulted in 4.31 g of the *E* isomer of POBC-H (yield: 87 %). ^1^H NMR ([D_6_]DMSO): *δ* = 7.71 (d, 2 H, Ph), 7.94 (d, 2 H, Ph), 8.21 (s, 1 H, C*H*=N), 11.52 (br. s, 1 H, NO*H*), 12.99 (br. s, 1 H, O*H*) ppm. ^13^C NMR ([D_6_]DMSO): *δ* = 126.40 (Ph), 129.66 (Ph), 131.11 (*C*–C=NOH), 137.16 (*C*–COOH), 147.51 (*C*=N), 166.90 ppm (*C*OOH) ppm.

***p*-[(3-Hydroxy-1-methyl-2-butenylidene)amino]benzoic Acid (PSBCA-H):** Acetylacetone (3.1 g, 31 mmol) was dissolved in ethanol (10 mL) and then added into a solution of *p*-aminobenzoic acid (4.1 g, 30 mmol) in ethanol (40 mL). The solution was heated at reflux for 5 h, and a clear yellow solution was obtained. The solution was kept at room temperature for crystallization. The light yellow crystals of PSBCA were isolated from the mother liquor, washed by diethyl ether, and dried at 70 °C; yield 5.26 g (80 %). ^1^H NMR ([D_6_]DMSO): *δ* = 2.02 (s, 3 H, N=C–C*H*_3_), 2.14 [s, 3 H, C(OH)C*H*_3_], 5.33 (s, 1 H, C*H*), 7.26 (d, 2 H, Ph), 7.90 (d, 2 H, Ph), 12.61 [s,1 H, C(CH_3_)O*H*], 12.81 (br. s, 1 H, COO*H*) ppm. ^13^C NMR ([D_6_]DMSO): *δ* = 19.85 (N=C–*C*H_3_), 29.13 [C(OH)*C*H_3_], 99.28 (*C*H), 122.14 (Ph), 126.27 (*C*–N=C), 130.59 (Ph), 142.69 (*C*–COO), 158.38 (*C*=N), 166.72 (*C*OOH), 195.98 [*C*(CH_3_)OH] ppm.

***p*-Salicyliden-*p*-aminobenzoic Acid (PSBC-H):** 2-Hydroxybenzaldehyde (3.8 g, 31 mmol) was dissolved in ethanol (10 mL), and then added into a solution of *p*-aminobenzoic acid (4.1 g, 30 mmol) in ethanol (40 mL). A yellow solid precipitated immediately. After the addition of ethanol (40 mL), the reaction mixture was heated at reflux for 5 h. Then the precipitate was filtered, washed with several portions of ethanol, and recrystallized from methanol. Dark yellow, needle-shaped crystals of PSBC-H were isolated, washed with diethyl ether, and dried at 70 °C; yield 4.19 g (58 %). ^1^H NMR ([D_6_]DMSO): *δ* = 12.97 (br. s, 1 H, COO*H*), 12.71 (br. s, 1 H, C_6_H_4_O*H*), 9.00 (s, 1 H, N=C*H*), 8.04 (d, 2H), 7.72 (d, 1 H), 7.51 (d, 2H), 7.42 (d, 1 H), 7.04 (t, 2 H) ppm. ^13^C NMR ([D_6_]DMSO): *δ* = 166.82 (*C*OOH), 164.74 (*C*H=N), 160.27 (*C*_Ph_OH), 152.13, 133.78, 132.57, 130.68, 128.76, 121.48, 119.26, 116.65 (C_Ph_) ppm.

***p*-Salicyliden-*p*-aminoacetophenone Oxime (PSBO-H):** A solution of salicylaldenhyde (3.17 g, 26 mmol) in ethanol (50 mL) was added dropwise to a solution of *p*-aminoacetophenone oxime (PABOM; 3.75 g, 25 mmol) in absolute ethanol (30 mL). The mixture was heated to 60 °C with stirring, and a yellow solid precipitated. After stirring overnight, the precipitate was filtered while hot, washed with cold ethanol and diethyl ether, recrystallized from acetone/water, and dried at 70 °C to a constant weight; yield 3.36 g (61 %). ^1^H NMR ([D_6_]DMSO): *δ* = 13.03 (s, 1 H, C–O*H*), 11.26 (s, 1 H, N–O*H*), 8.98 (s, 1 H, N=C*H*), 7.71 (d, 2 H, Ph), 7.64 (d, 1 H, C_6_H_4_), 7.44 (d, 2 H, C_6_H_4_), 7.38 (d, 1 H, C_6_H_4_), 6.98 (t, 2 H, C_6_H_4_), 2.16 (s, 3 H, C*H*_3_) ppm. ^13^C NMR ([D_6_]DMSO): *δ* = 163.4 (N=*C*H), 160.3 (*C*–OH), 152.4 (*C*=N), 148.1, 135.5, 133.4, 132.5, 126.6, 121.4, 119.3, 119.1, 116.6 (C_6_H_4_), 11.4 (*C*H_3_) ppm.

**Zn(POBC)_2_:** A solution of Zn(OAc)_2_**·**2H_2_O (1.09 g, 5 mmol) in ethanol (20 mL) was added to a solution of POBC-H (10 mmol) in ethanol (40 mL) at 50 °C. The solution became clear after stirring for 2 h. The solvent was then removed under reduced pressure, and a white powder was obtained. The powder was extracted several times with diethyl ether and then dried at 120 °C to constant weight; yield 1.76 g (89 %). ^1^H NMR ([D_6_]DMSO): *δ* = 7.63 (d, 2 H, Ph), 7.94 (d, 2 H, Ph), 8.18 (s, 1 H, C*H*=N), 11.37 (br. s, 1 H, O*H*) ppm. ^13^C NMR ([D_6_]DMSO): *δ* = 125.91 (Ph), 129.83 (Ph), 135.32 (*C*–COO), 147.80 (*C*=N), 171.10 (*C*OO) ppm. C_16_H_12_N_2_O_6_Zn (393.66): calcd. C 48.77, H 3.05, N 7.11; found C 47.47, H 3.32, N 6.05. TGA weight loss: calcd. (%) for ZnO 79.3; found 80.1.

**Zn(PSBCA)_2_:** A solution of Zn(OAc)_2_**·**2H_2_O (1.09 g, 5 mmol) in ethanol (20 mL) was added to a hot solution of PSBCA-H (10 mmol) in ethanol (40 mL) at 50 °C. A colorless solid precipitated immediately. After further stirring for 2 h, the precipitate was filtered while hot, washed with cold ethanol and diethyl ether, and then dried at 120 °C to constant weight; yield 1.57 g (62 %). ^1^H NMR ([D_6_]DMSO): *δ* = 2.01 (s, 3 H, N=C–C*H*_3_), 2.10 [s, 3 H, C(OH)C*H*_3_], 5.29 (s, 1 H, C*H*), 7.20 (d, 2 H, Ph), 7.93 (d, 2 H, Ph), 12.58 (s,1 H, O*H*) ppm. ^13^C NMR ([D_6_]DMSO): *δ* = 19.72 (N=C–*C*H_3_), 29.05 [C(OH)*C*H_3_], 98.54 (*C*H), 122.22 (Ph), 130.68 (Ph), 140.95 (*C*–COO), 158.96 (*C*=N), 170.87 (*C*OO), 195.53 [*C*(OH)CH_3_] ppm. C_24_H_24_N_2_O_6_Zn (501.84): calcd. C 57.39, H 4.78, N 5.58; found C 56.96, H 4.92, N 5.42. TGA weight loss: calcd. (%) for ZnO 83.8; found 82.7.

**Reaction of Zinc Acetate with PSBC-H:** The procedure was the same as for the synthesis of Zn(PSBCA)_2_. A yellow precipitate was obtained; yield 1.81 g (66 %). ^1^H NMR ([D_6_]DMSO): *δ* = 12.89 (br. s, 1 H, C_Ph_–O*H*), 9.00 and 8.71 (s, 1 H, N=C*H*), 8.00 (d) and 7.7–6.5 ppm (6 H, C_6_*H*_4_) ppm. ^13^C NMR ([D_6_]DMSO): *δ* = 170.98 (*C*OO), 164.20 and 168.88 (*C*=N), 160.28 (*C*_Ph_OH), 150.93 (*C*_Ph_N), 137.5–116.0 (C_6_H_4_) ppm. C_56_H_38_N_4_O_12_Zn_2_ [Zn(PSBC)_2_]: calcd. C 61.55, H 3.48, N 5.13; found C 59.25, H 3.65, N 5.01. TGA weight loss: calcd. (%) for ZnO 85.1; found 85.0.

**Zn_2_(OAc)(OOC–C_6_H_4_–NH_2_)_3_:** Zn(OAc)_2_**·**2H_2_O (0.55 g, 2.5 mmol) was added to a solution of PABC-H (1.37 g, 10 mmol) in ethanol (20 mL). After stirring for 10 min, a colorless solid precipitated. The solution was sealed and kept for 4 weeks, during which time the precipitate transformed into light yellow crystals; yield 0.32 g (43 %). ^1^H NMR ([D_6_]DMSO): *δ* = 7.64 (d, 2 H, Ph), 6.51 (d, 2 H, Ph), 5.51 (s, 2 H, N*H*_2_), 1.82 ppm (s, 1 H, C*H*_3_) ppm.

**Zn(PSBO)_2_:** The procedure was the same as for the synthesis of Zn(PSBCA)_2_. A light yellow precipitate was obtained; yield 1.74 g (61 %). ^1^H NMR ([D_6_]DMSO): *δ* = 11.21 (s, 1 H, O*H*), 8.80 (s, 1 H, N=C*H*), 7.60 (d, 2 H), 7.50 (d, 1 H), 7.37 (d, 1 H), 7.31 (d, 2 H), 6.73 (d, 1 H), 6.66 (d, 1 H), 2.09 (s, 3 H, C*H*_3_) ppm. ^13^C NMR ([D_6_]DMSO): *δ* = 170.8 (N–*C*H), 170.1 (*C*–OH), 152.1 (*C*=N), 148.5, 137.5, 135.9, 135.4, 126.8, 122.7, 121.3, 118.7, 114.6 (C_6_H_4_), 11.3 (*C*H_3_) ppm. C_30_H_26_N_4_O_4_Zn (571.94): calcd. C 62.94, H 4.55, N 9.79; found C 63.23, H 4.58, N 9.73. TGA weight loss: calcd. (%) for ZnO 85.8; found 85.3.

**X-ray Structure Analysis:** Single-crystal X-ray diffraction experiments were performed at 100 K with a Bruker-AXS SMART APEX II diffractometer with a CCD area detector and a crystal-to-detector distance of 5.0 cm using graphite-monochromated Mo-*K*_α_ radiation (*λ* = 71.073 pm). Data were collected with *φ* and *ω* scans and a 0.5° frame width. The data were corrected for polarization and Lorentz effects, and an empirical absorption correction (SADABS^16^) was employed. The cell dimensions were refined with all unique reflections. SAINT PLUS software^17^ was used to integrate the frames. Details of the X-ray investigations are given in Table 1.

**1:** Crystallographic data for [Zn_2_(OAc)(OOC–C_6_H_4_–NH_2_)_3_]_∞_.

Empirical formula	C_48_H_48_N_6_O_17_Zn_4_
*M*_r_	1242.4
Crystal system	monoclinic
Space group	*C*2/*c*
*a* [pm]	2407.0(1)
*b* [pm]	1417.02(7)
*c* [pm]	1852.8(1)
*β* [°]	126.991(2)
*V* [pm^3^]**·**10^6^	5047.4(5)
*Z*	4
*D*_calcd._ [Mg m^–3^]	1.635
*μ* [mm^–1^]	1.957
Crystal size [mm]	0.20 × 0.20 × 0.20
Measured, unique reflections	21773, 4455
*θ*_max_ [°]	25.00
*R*^1^, *wR*^2^ [*I* < 2*σ*(*I*)]	0.0464, 0.1631
Reflections/parameters	4455/384
Weighting scheme	*w* = 1/[*σ*^2^(*F*_0_^2^) + (0.1146*P*)^2^ + 16.3637*P*[a]
*δρ*_max._, *δρ*_min_ [e Å^–3^]	3.481, –0.466

[a] *P* = (*F*_o_^2^ +2*F*_c_^2^)/3.

The structures were solved by direct methods (SHELXS97^18^). Refinement was performed by the full-matrix least-squares method on the basis of *F*^2^ (SHELXL97) with anisotropic thermal parameters for all non-hydrogen atoms. Hydrogen atoms were inserted in calculated positions and refined riding with the corresponding atom; those bonded to oxygen and nitrogen atoms were identified in the electron density map.

CCDC-883699 contains the supplementary crystallographic data for this paper. These data can be obtained free of charge from The Cambridge Crystallographic Data Centre via www.ccdc.cam.ac.uk/data_request/cif.

**Supporting Information** (see footnote on the first page of this article): ^1^H and ^13^C NMR spectra, as well as IR spectra and TGA of all complexes.
