# Supplementary material for: Zinc(II) Complexes with Dangling Functional Organic Groups
Source: Eur J Inorg Chem. 2012 Aug 16;2012(27):4294–300. doi: 10.1002/ejic.201200558 (PMC4038256; doi:10.1002/ejic.201200558)
Supplement: Supplementary file 1 [file ejic2012-4294-SD1.pdf]

Eur. J. Inorg. Chem. **2012** · © WILEY-VCH Verlag GmbH & Co. KGaA, 69451 Weinheim, 2012 · ISSN 1434–1948

SUPPORTING INFORMATION

DOI: 10.1002/ejic.201200558
Title: Zinc(II) Complexes with Dangling Functional Organic Groups
Author(s): Jingxia Yang, Michael Puchberger, Renzhe Qian, Christian Maurer, Ulrich Schubert*

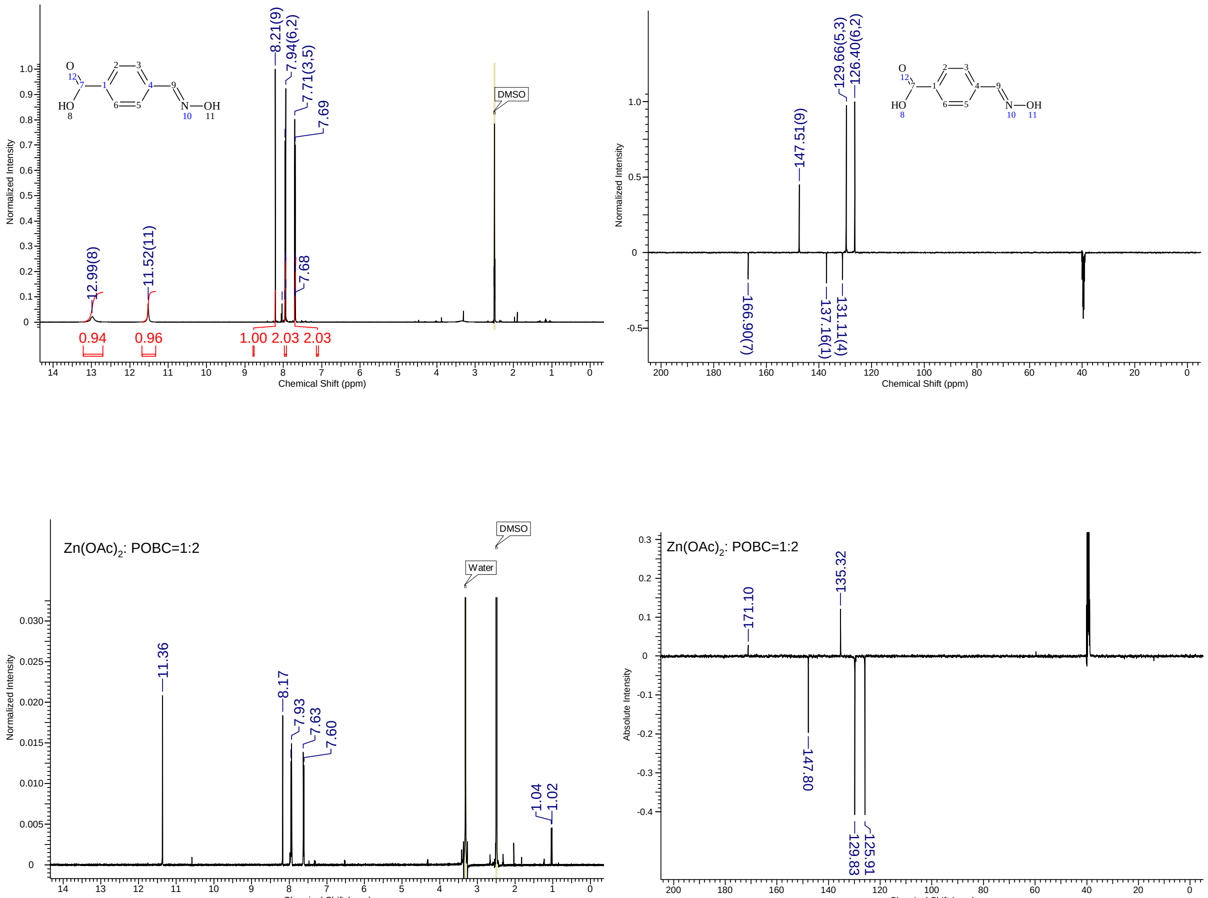

Figure S1. ^{1}H and ^{13}C NMR spectra of POBC (top) and $Zn(POBC)_2$ (bottom).

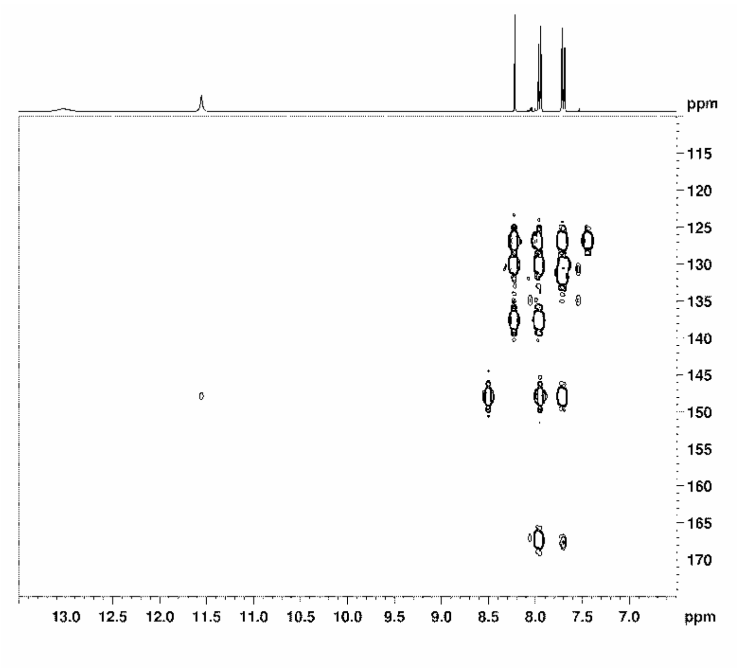

Figure S2. HMBC spectrum of POBC in d_6-DMSO

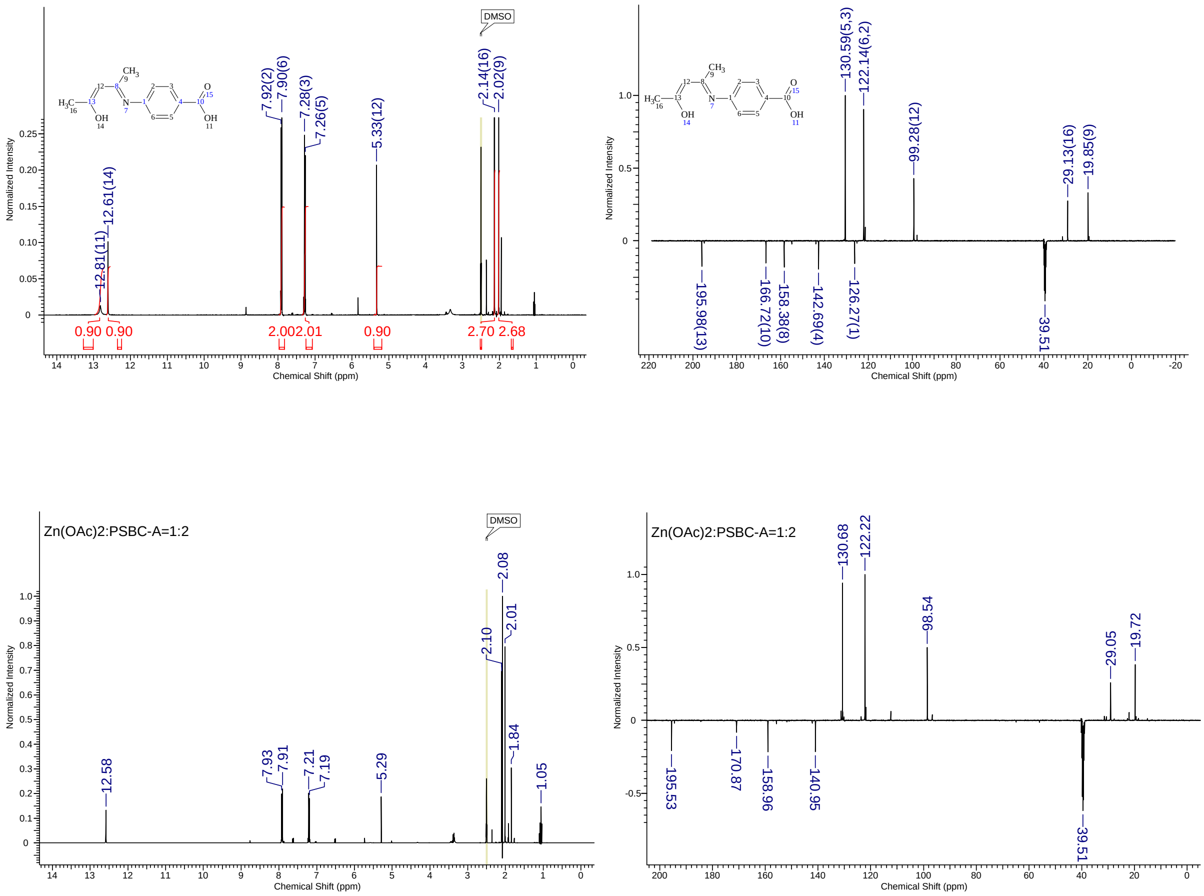

Figure S3. ^1H and ^{13}C NMR spectra of PSBCA-H (top) and $Zn(PSBCA)_2$ (bottom).

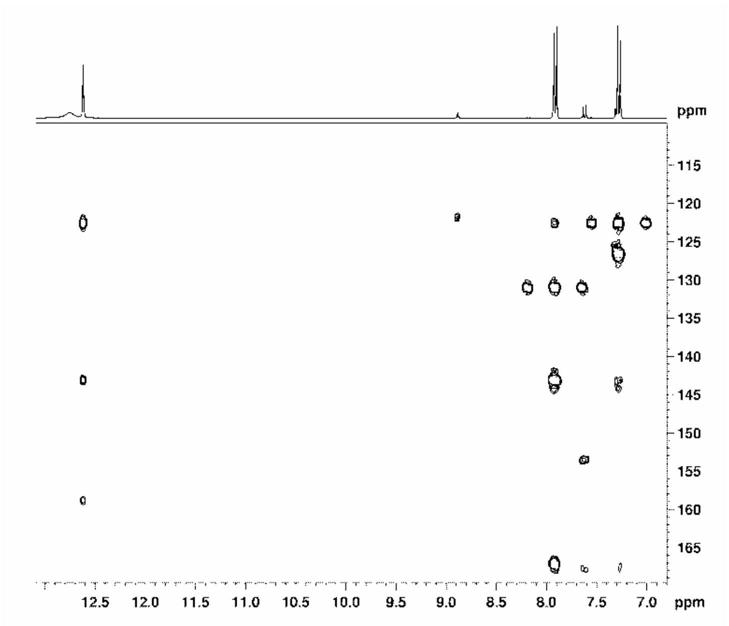

Figure S4. HMBC spectrum of PSBCA-H in d_6-DMSO

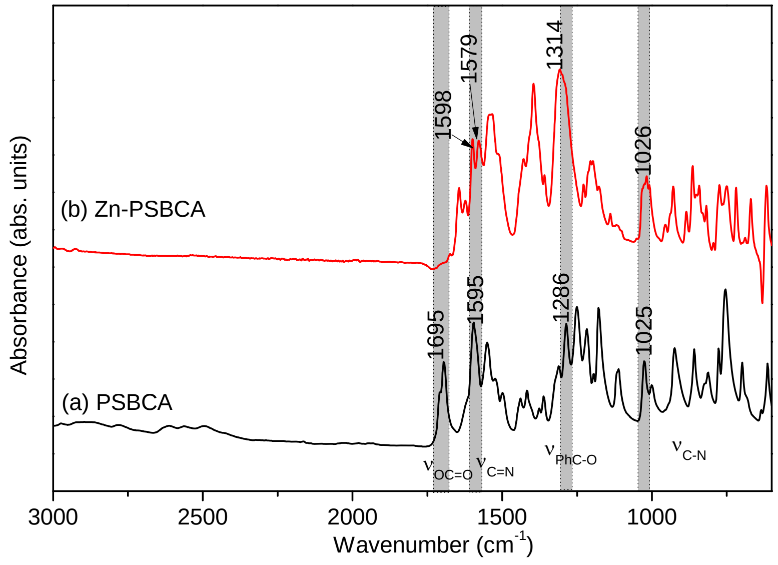

Figure S5. FT-IR spectra of the PSBCA-H and $Zn(PSBCA)_2$.

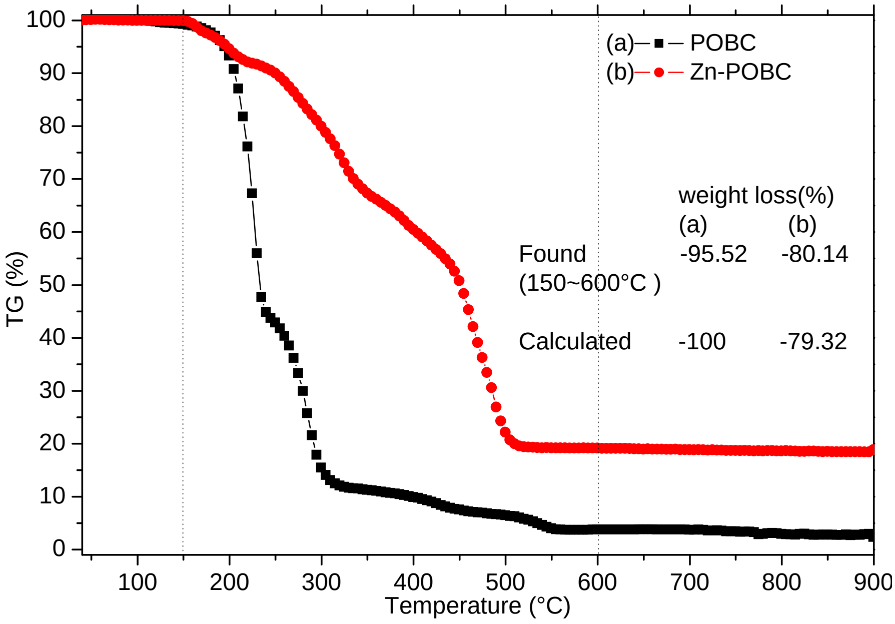

Figure S6. TGA of POBC and Zn(POBC)$_2$.

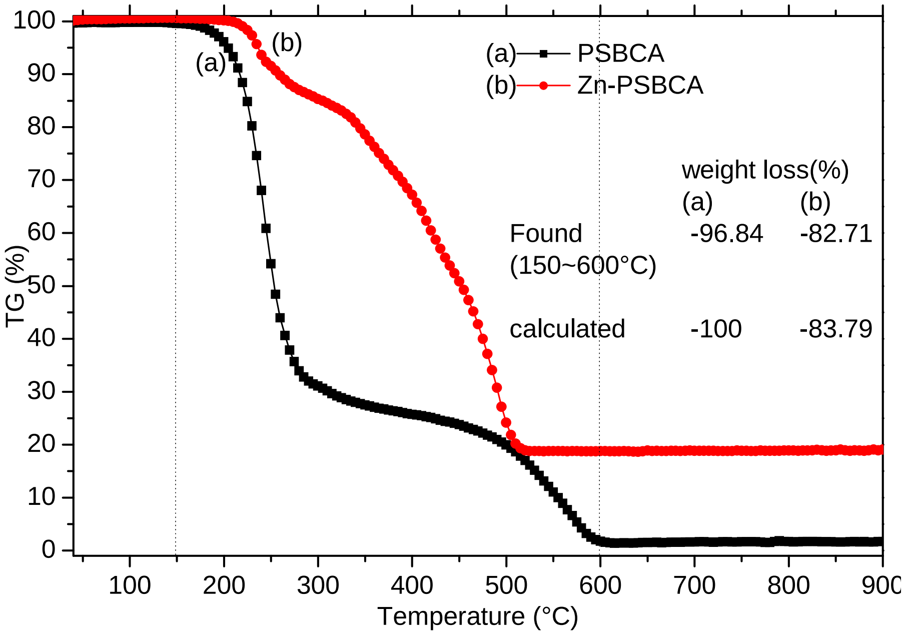

Figure S7. TGA of PSBCA-H and Zn(PSBCA)$_2$.

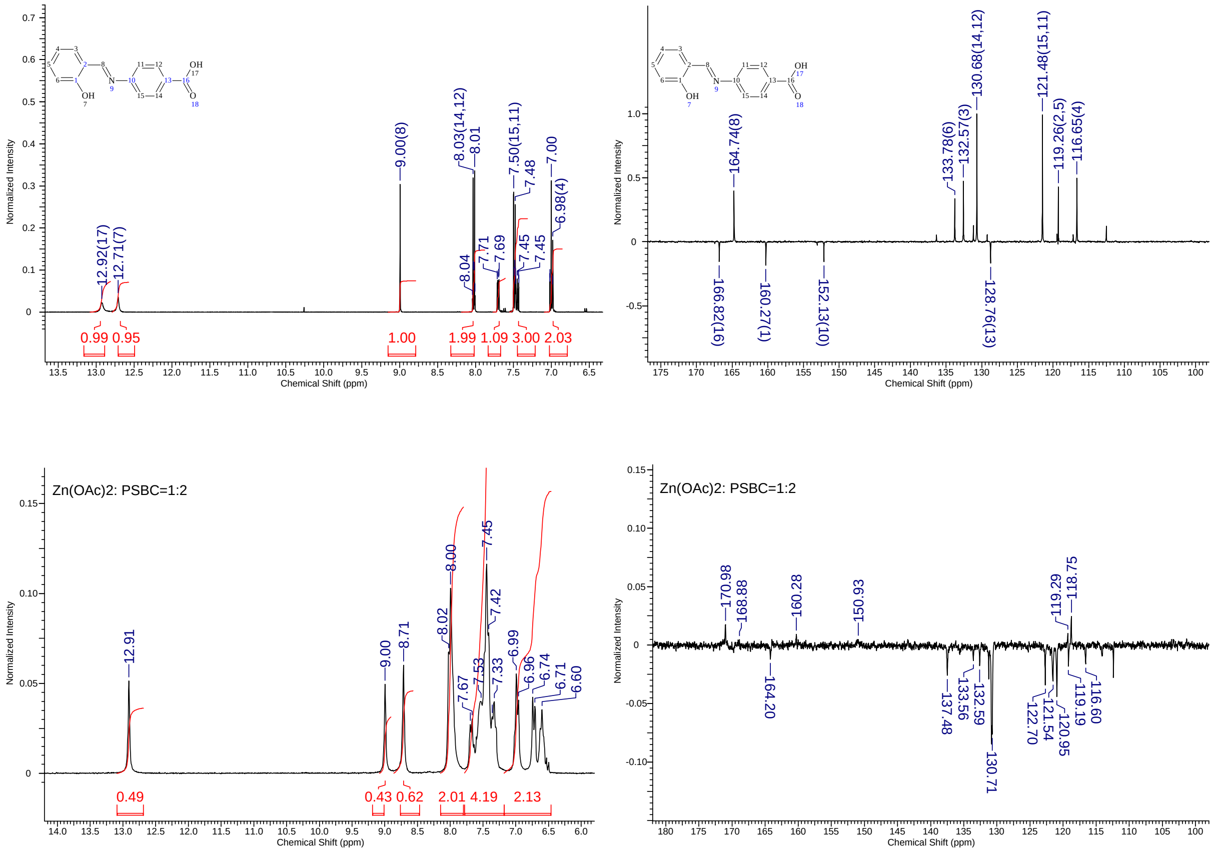

Figure S8. ^{1}H and ^{13}C NMR spectra of PSBC-H (top) and $Zn(PSBC)_2$ (bottom).

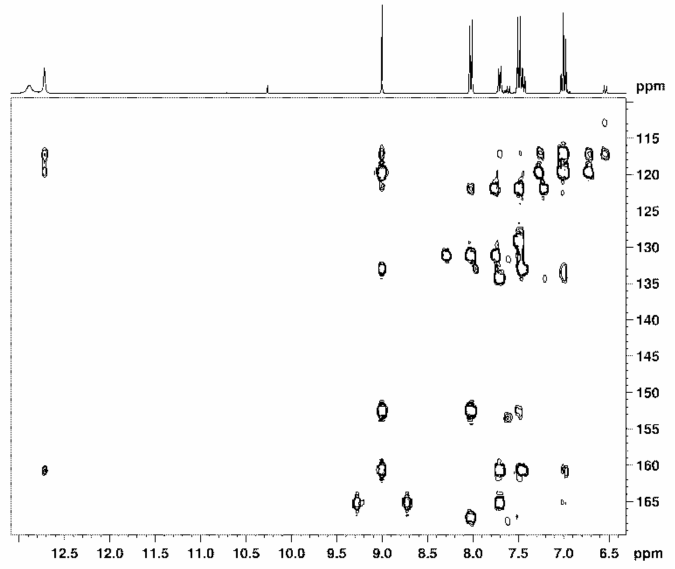

Figure S9. HMBC spectrum of PSBC-H in d_6-DMSO

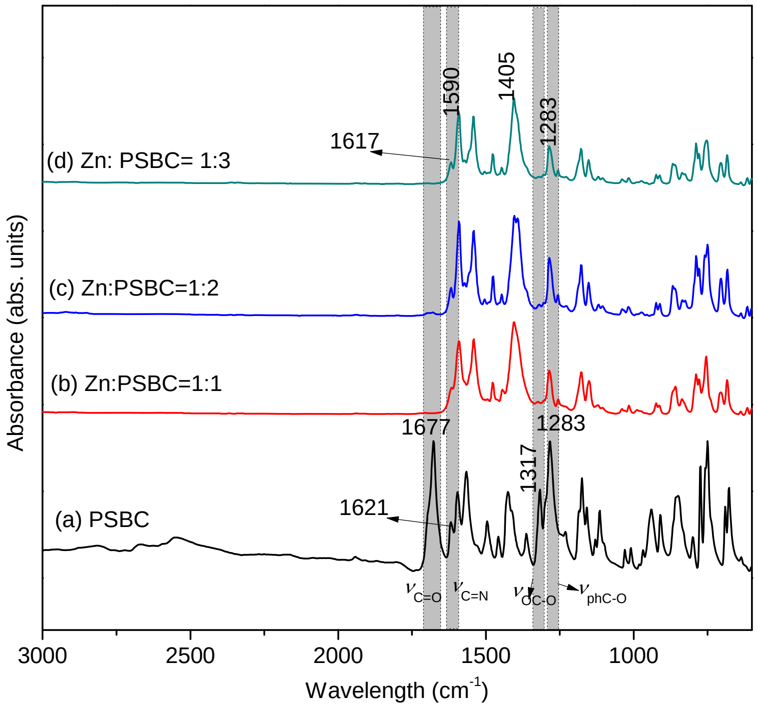

Figure S10. FT-IR spectra of PSBC and Zn-PSBC complexes.

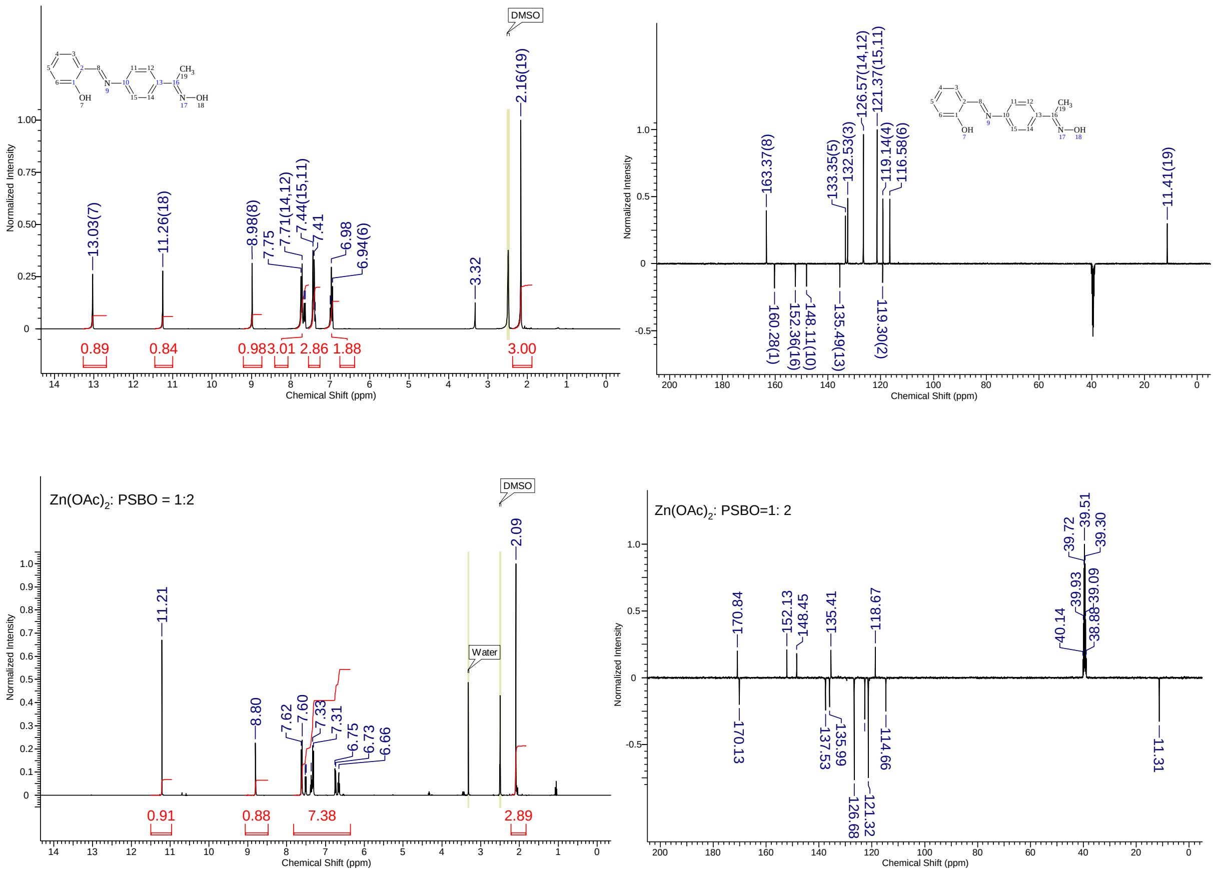

Figure S11. ^{1}H and ^{13}C NMR spectra of PSBO-H (top) and $Zn(PSBO)_2$ (bottom).

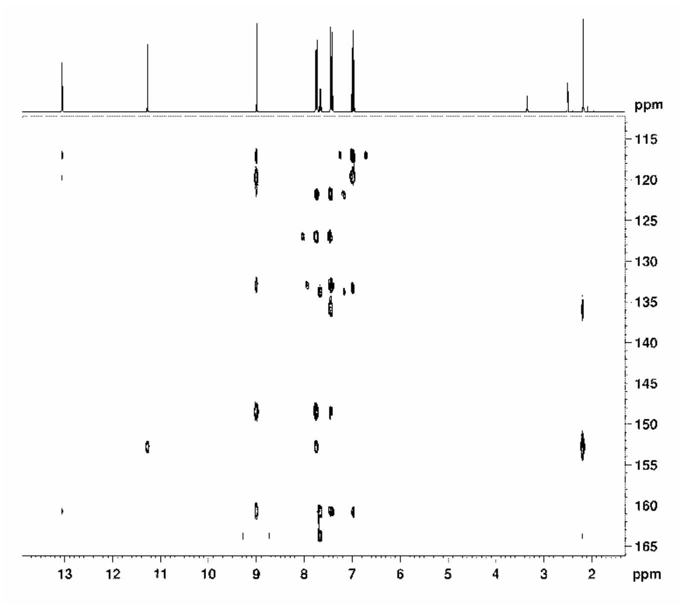

Figure S12. HMBC spectrum of PSBO-H in d_6-DMSO

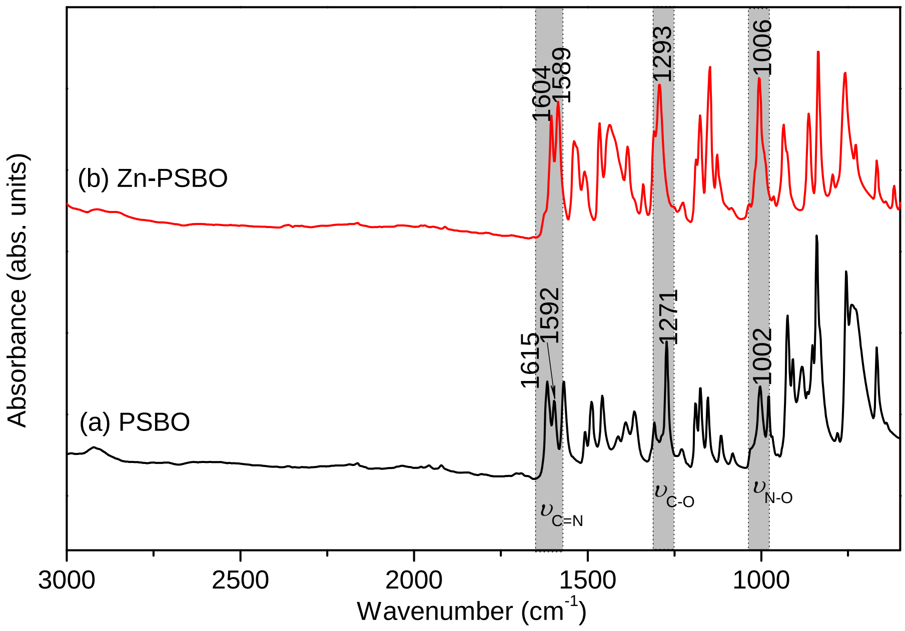

Figure S13. FT-IR spectra of PSBO-H and $Zn(PSBO)_2$.

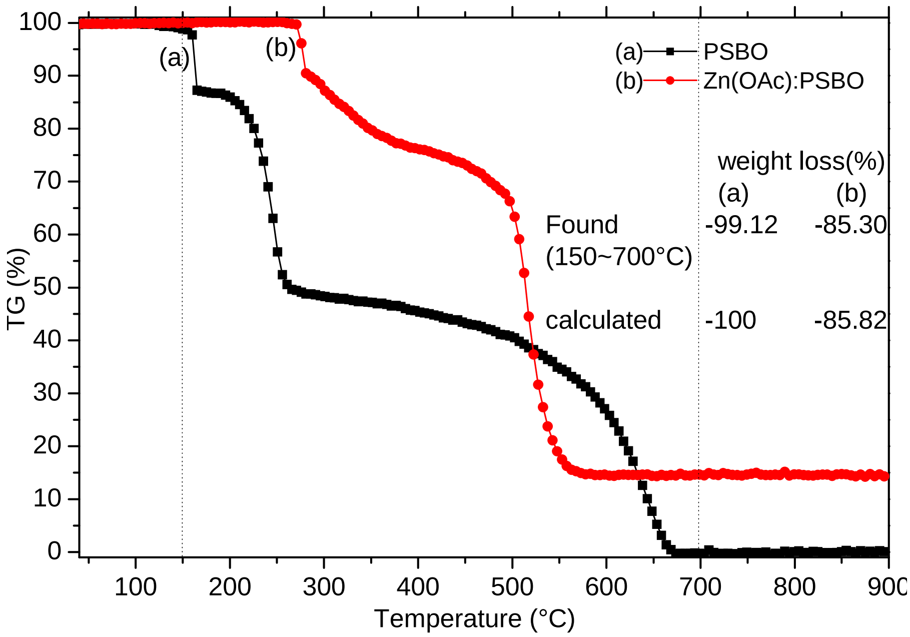

Figure S14. TG curves of the PSBO and Zn-PSBO complex
